# Eikenella corrodens endocarditis and liver abscess in a previously healthy male, a case report

**DOI:** 10.1186/s12879-018-2949-4

**Published:** 2018-01-12

**Authors:** Anne Christine Nordholm, Ruth Ottilia Birgitta Vøgg, Henrik Permin, Terese Katzenstein

**Affiliations:** 1 Department of Infectious Diseases, Copenhagen University Hospital, Rigshospitalet, Copenhagen, Denmark; 2 Department of Respiratory Medicine, Bispebjerg Hospital, Copenhagen, Denmark; 3 Department of Cardiology, Copenhagen University Hospital, Rigshospitalet, Copenhagen, Denmark

**Keywords:** *Eikenella corrodens*, HACEK, Liver abscess, Endocarditis, Case report

## Abstract

**Background:**

*Eikenella corrodens* is one of the HACEK bacteria constituting part of the normal flora of the oropharynx, however, still an uncommon pathogen. We report a case of a large *Eikenella corrodens* liver abscess with simultaneously endocarditis in a previously healthy male.

**Case presentation:**

A 49-year-old Danish man was admitted because of one-month malaise, fever, cough and unintentional weight loss. On admission there was elevated white blood cell count and C-reactive protein, as well as affected liver function tests. Initially pneumonia was suspected, but due to lack of improvement on pneumonia treatment, a PET-CT scan was performed, which showed a large multiloculated abscess in the liver. The abscess was drained using ultrasound guidance. Culture demonstrated *Eikenella corrodens*. Transesophageal echocardiography revealed aortic endocarditis. The patient was treated with antibiotics and abscess drainage, on which he slowly improved. He was discharged after 1.5 months of hospitalisation. On follow-up 2 months later, the patient was asymptomatic with normalized biochemistry and ultrasound showed complete regression of the abscess.

**Conclusions:**

This is the first case of documented *Eikenella corrodens* concurrent liver abscess and endocarditis. The case report highlights that *Eikenella corrodens* should be considered as a cause of liver abscess. Empirical treatment of pyogenic liver abscess will most often cover *Eikenella corrodens,* but the recommended treatment is a third generation cephalosporin or a fluoroquinolon. A multiloculated liver abscess may require drainage several times during treatment. The finding of *Eikenella corrodens* should elicit an echocardiography to diagnose endocarditis even in patients without clinical signs of endocarditis.

## Background

The HACEK group of bacteria includes *Haemophilus parainfluenzae, Aggregatibacter* spp.*, Cardiobacterium* spp.*, Eikenella corrodens* and *Kingella* spp. [1]. These are fastidious gram-negative bacteria that are part of the normal human mucosal flora, predominantly of the oropharynx [2]. HACEK bacteria have traditionally been associated with endocarditis and causes approximately 3% of all infective endocarditis [3], but blood cultures are frequently sterile in HACEK endocarditis cases [4]. *Eikenella corrodens* is the least common cause of HACEK endocarditis and it has only been sporadically described in the literature and often in patients with risk factors [4]; in particular recent dental therapy, intravenous drug abuse, immunosuppression or valvular damage [5]. In the 1970ies it was discovered that *Eikenella corrodens* could cause visceral abscesses including liver abscesses [6]. Cases of *Eikenella corrodens* causing liver abscesses have only been reported 12 times in the literature previously [6–17]. Pyogenic liver abscesses (PLA) may result from haematogenous seeding from the systemic circulation, gallstones or malignancies and risk factors include hepatobiliary infection, pancreatic disease, diabetes and prior liver transplant [18]. Treatment often requires both catheter drainage and long-term antibiotic treatment [19]. Here, we report a unique case of *Eikenella corrodens* endocarditis and a large liver abscess in a previously healthy adult male.

## Case presentation

### Admission

A 49-year-old Caucasian male with an unremarkable medical history presented to the emergency department on a local hospital due to a four-week history of malaise, fever, cough and an unintended weight loss of 7 kg. He had no recent travel history or history of drug abuse. Based on the symptoms and stethoscopic findings pneumonia was suspected and the patient was admitted to the Department of Respiratory Diseases. On admission his blood pressure was 135/67 mmHg, heart rate 125 beats per minute, temperature 36,4 °C, and with an oxygen saturation of 92% with 2 l of oxygen supplementation. On auscultation there were crackles at the right lung, but further clinical examination was unremarkable and he was at no point in acute distress.

### Investigations

**Fig. 1:**
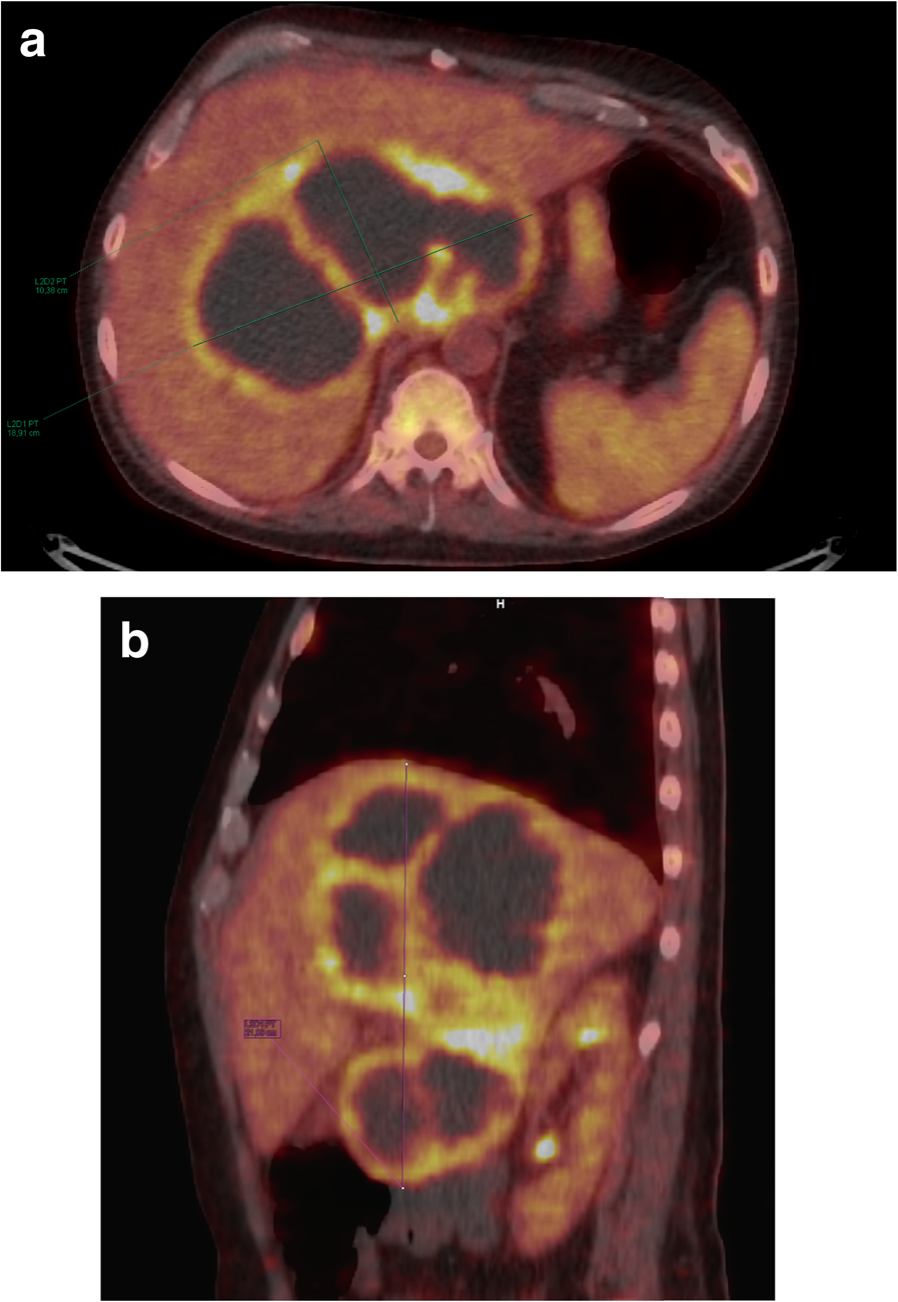
**a** Transaxial PET-CT scan demonstrating the large multiloculated liver abscess measuring 10.38 × 18.98 cm. **b** Sagittal PET-CT scan showing the large multiloculated liver abscess measuring 21,99 cm in cranio-caudal dimension

**Fig. 2:**
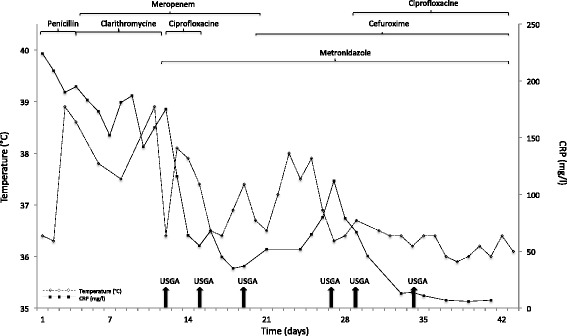
Treatment with antibiotics and ultrasound-guided aspirations (USGA, vertical arrows) in relation to temperature and CRP from admission to discharge

Laboratory examination revealed elevated white blood cell count (WBC) at 20 × 10^9^/L and C-reactive protein (CRP) at 224 mg/L along with increased liver function tests (Alanine aminotransferase at 84 U/L and Alkaline phosphatase at 256 U/L) and hypoalbuminemia at 19 g/L. Chest radiograph showed no obvious infiltrates. Pharyngeal swabs and sputum cultures were negative. Blood and urine cultures came out negative, though collected before the antibiotic treatment was commenced. The blood culture method used was BacT/Alert® an- and aerobic collection bottles incubated 5 days and later BD BACTEC Plus an- and aerobic Culture Vials incubated for 7 days. We tested for hepatitis infection; Immunoglobulin (Ig) M for hepatitis A, IgG surface antibody and surface antigen for hepatitis B, IgG for hepatitis C and IgM and IgG for hepatitis E virus (HEV). Of these HEV IgG was positive while the rest all came out negative. In addition, serology tests were negative for Epstein-Barr virus, cytomegalovirus, human immunodeficiency virus, *Pneumocystis jirovecii* and *Entamoeba histolytica*. In spite of antibiotic treatment for more than a week (Fig. 2) the patient did not improve, which led to performance of a positron emission tomography-computer tomography (PET-CT) scan in search for the focus of infection. The scan revealed a massive multiloculated abscess (22x18cm) (Fig. 1a and b), along with cholelithiasis (1x1cm) and lymph nodes enlargement. The following ultrasound-guided abscess catheterisation resulted in drainage of 500 mL of pus and a pig-tail catheter was left in situ*.*

After the finding of a liver abscess the patient was transferred to the Department of Infectious Diseases, Copenhagen University Hospital, Rigshospitalet. Abscess material was cultured both anaerobically and aerobically and from the latter grew *Eikenella corrodens*. Further blood cultures were collected but remained negative. Because of the finding of a HACEK bacterium, endocarditis was suspected. There were no valvular murmurs at the clinical examination and no other clinical signs of endocarditis, however transthoracic echocardiography showed an excrescence on the aortic valve. A subsequent transoesophageal echocardiography confirmed aortic endocarditis without valve insufficiency.

In the search for the origin of infection, a panoramic dental radiography was performed showing no signs of dental abscesses but extremely poor tooth status, leading to extraction of 10 teeth.

### Treatment

When diagnosed with PLA on PET-CT and after ultrasound-guided drainage, the patient was empirically treated with meropenem, ciprofloxacin and metronidazole. After recognition of the *Eikenella corrodens* resistance pattern, antibiotic treatment was narrowed to cefuroxime, and metronidazole was continued to cover other potential anaerobic pathogens. Due to a new onset of fevers and subsequent elevation of WBC and CRP, intravenous ciprofloxacin was reintroduced and further improvement was observed. The patient was treated with ultrasound-guided abscess aspirations six times (2–7 days intervals) and with pig-tail catheters in between in order to drain different parts of the multiloculated abscess. Antibiotic treatment and ultrasound-guided aspirations in relation to temperature and CRP level are illustrated in Fig. 2. In total, the patient was treated with intravenous therapy for 6 weeks to cover both liver abscess and endocarditis. After discharge, he received high-dose oral antibiotics with amoxicillin and ciprofloxacin for another 2 weeks.

### Out-come and follow up

The patient improved on the combination of antibiotics and continuous liver drainage, fever faded and his appetite returned. At discharge the WBC and CRP were within normal range, alanine aminotransferases was normalized. Alkaline phosphatase remained slightly elevated and he continued to have slight hypoalbuminemia 25 g/L. On follow-up in the outpatient clinic after 2 months the patient was well and asymptomatic with normalized biochemistry and almost complete regression of the liver abscess on ultrasound.

## Discussion and conclusions

This is the first case report in the literature of co-occurrence of *Eikenella corrodens* endocarditis and liver abscess*.* The liver abscess is the largest ever described caused by this bacteria. Reviewing the literature revealed only 12 previous case reports of *Eikenella corrodens* liver abscess [6–17] and 18 cases of endocarditis [5, 20–37]. The opportunistic pathogen *Eikenella corrodens* is one of the HACEK bacteria and constitute part of the normal human mucosal flora, predominantly the oropharynx [2]. In this case report, the focus of infection was most likely the oropharynx, as the patient’s dental hygiene was very poor. We never isolated the bacteria from the blood, which is not uncommon neither in the case of liver abscesses [38] nor with HACEK endocarditis [4]. Usually, *Eikenella corrodens* infections are described as part of polymicrobial infections [39], but despite thorough culturing, no other microorganisms were isolated from either pus or blood in our patient.

Patients with PLA often present with fever, nausea and abdominal pain, however it is not uncommon with an unremarkable clinical examination and negative blood cultures [38], which challenges the diagnostic. Our patient had a protracted disease course with malaise, fever, cough and an unintentional weight loss of 7 kg, but never any abdominal pain, and the diagnosis of the liver abscess was not determined until a PET-CT scan was performed. Most of the other case reports of *Eikenella corrodens* liver abscesses describe a more acute onset of symptoms [9–17]. In contrast *Eikenella corrodens* endocarditis has been described with a insidious disease course [1] and the time for diagnosis has varied from 1 to 4 months [22].

We treated our patient with broad-spectrum antibiotics after the identification of the liver abscess and he responded well, but when trying to narrow the therapy, WBC and CRP increased, the patient turned febrile, and ciprofloxacin was reintroduced after which the patient improved. *Eikenella corrodens* is typically resistant to metronidazole, clindamycin and aminoglycosides [17] but susceptible to cephalosporins and fluoroquinolons which is the recommended treatment in *Eikenalla corrodens* infections. There has been described large variations between the different generations of the cephalosporins with third generations being much more potent against *Eikenella corrodens* infections than second and first generations [40]. The observed improvement after reintroduction of ciprofloxacine most likely illustrates that cefuroxime alone is not so potent and that a combination with a fluoroquinolone is a better choice. In order to cover anaerobic pathogens our patient received metronidazole, as other case reports have also suggested [12]. The antibiotic therapy was decided in order to treat both endocarditis and the liver abscess, though the latter needed supplementary drainage. In most previous case reports drainage has only been necessary for a few days, whereas our patients needed a combination therapy with both continuous drainage and aspirations with 2–7 days intervals for almost 1 month on which the abscess decreased in size. To our knowledge our patient had the largest multiloculated abscess with *Eikenlla corrodens* ever described. In the current literature, *Eikenella corrodens* has never been described as the cause of both liver abscess and endocarditis, which, therefore, must be considered particularly rare.

Despite of limited pathogenicity, *Eikenella corrodens* should be considered as a potential pathogen causing liver abscess and a multiloculated lever abscess may require drainage several times and pus should be examined as blood cultures might come out negative. Investigations should include dental inspection and imaging of the abdomen. The finding of *Eikenella corrodens,* a member of the HACEK group, should cause investigation of endocarditis even in previously healthy patients without obvious clinical signs of endocarditis.

## Abbreviations

CRP: C-reactive protein
Ig: Immunoglobulins
PET-CT: Positron emission tomography-computer tomography
PLA: Pyogenic liver abscess
USGA: Ultrasound-guided aspirations
WBC: White blood cell count
